# Individual Phenotype Does Not Impact the Outcome of Mechanical Aligned Total Knee Arthroplasties for Valgus Osteoarthritis

**DOI:** 10.3390/medicina59101852

**Published:** 2023-10-18

**Authors:** Laura E. Streck, Martin Faschingbauer, Marco Brenneis, Cosima S. Boettner, Kilian List, Maximilian F. Kasparek, Friedrich Boettner

**Affiliations:** 1 Adult Reconstruction and Joint Replacement Department, Hospital for Special Surgery, 535 East 70th Street, New York, NY 10021, USA; 2 Department of Orthopedic Surgery, University of Ulm, Oberer Eselsberg 45, 89081 Ulm, Germany; 3 The Complex Joint Reconstruction Centre at Hospital for Special Surgery, 535 East 70th Street, New York, NY 10021, USA; 4 Department of Orthopedics (Friedrichsheim), University Hospital Frankfurt, Goethe University, 60590 Frankfurt am Main, Germany; 5 Department of Orthopedics, University of Wuerzburg, Brettreichstrasse 11, 97074 Wuerzburg, Germany; 6 Department of Orthopedics, Evangelisches Krankenhaus, Hans-Sachs Gasse 10-12, 1180 Vienna, Austria

**Keywords:** mechanical alignment, kinematic alignment, CPAK, valgus osteoarthritis, valgus knee, phenotype

## Abstract

*Background and Objectives*: There is an ongoing discussion about the best alignment targets in total knee arthroplasty (TKA). Mechanical alignment has been the standard in TKA for years. Alongside the development of various classification systems to describe the native alignment of the knee (knee phenotype), kinematic alignment restoring the individual phenotype of the knee has been advocated more recently. Alignment in TKA becomes even more challenging in knees with preoperative deformities such as valgus osteoarthritis. *Materials and Methods*: The study retrospectively evaluated 158 knees in 135 patients who underwent TKA with a mechanical alignment target for valgus osteoarthritis. Pre- and postoperative hip knee angle, lateral distal femur angle, and medial proximal tibial angle/tibial plate angle (pre-/postoperative) were measured on standing hip-to-ankle radiographs. Knees were grouped according to the coronal plane alignment of the knee (CPAK) classification. Preoperative and postoperative range of motion and patient-related outcome measures (WOMAC, UCLA, SF-12, pain) were assessed. *Results*: There was no difference in outcome for mechanically aligned TKA between the different CPAK phenotypes, suggesting that mechanical alignment is an appropriate target for the different phenotypes analyzed in the study. Remaining valgus alignment was associated with decreased postoperative UCLA scores and decreased improvement in SF-12 scores (*p* = 0.011/*p* = 0.028). Within CPAK III, mechanical aligned TKA showed better postoperative UCLA Scores than TKA with valgus alignment (*p* = 0.015). The individual knee phenotype in patients with valgus osteoarthritis did not influence the outcome of mechanical aligned TKA operated with standardized soft-tissue release.

## 1. Introduction

Ever since the development of total knee arthroplasties (TKAs), the alignment of the components has been considered an important factor for implant function and longevity. Yet the optimum targets for TKA alignment are highly controversial and under ongoing debate. The most common alignment technique is neutral component alignment or mechanical alignment (MA). A neutral mechanical alignment of the lower extremity is defined as a line from the hip center through the knee center through the ankle center. The cut angles are determined by the difference between the femoral and tibial anatomical and mechanical axes. Hereby, the components can be positioned perpendicular to the mechanical axis of the lower extremity. Flexion and extension gaps are balanced subsequently by soft-tissue release.

MA aims to reduce sheer and bending forces on the bone–prosthesis interface. These biomechanical considerations have been supported by finite element analyses showing lower contact stresses in neutral aligned knees [1]. The goal is to improve the longevity of the implant. MA has been the gold standard in TKA in the past [2,3,4].

In recent years, more studies have focused on the assessment of the original, individual alignment of the native knee (phenotype) and found large variations within the population [5,6]. There has been criticism that MA disregards this native phenotype of the knee.

Furthermore, there is a growing patient population not only seeking pain relief but also becoming more demanding regarding functionality and return to sports [7,8]. The focus in joint arthroplasty surgery is increasingly on meeting these expectations, and longevity defining successful TKA is only one amongst further criteria. This has led to doubts about whether a strictly mechanical alignment can do justice to each individual knee and patient.

It has been suggested that the mismatch between the native phenotype and the mechanical alignment might cause dissatisfaction, unexplained pain, and instability after TKA [4,9,10,11]. The effects of the alignment techniques become even more relevant with the more coronal deviation there is in the native knee.

Some authors have suggested that a kinematic alignment (KA) approach to restore the patient’s native alignment may be favorable [11,12,13]. Kinematic alignment by nature reduces the need for soft-tissue releases, yet it may bear the risk of imbalanced stresses on the implant. Furthermore, various techniques have been described to adequately release the soft-tissue even in knees with severe valgus osteoarthritis during MA TKA [14,15,16,17].

A meta-analysis of randomized controlled trials comparing MA and KA TKA using patient-specific implants by Woon et al. [18] showed no difference for early WOMAC and KSS combined scores as well as no differences between patients with native varus, valgus, or neutral alignment [18]. However, long term data on KA TKA, especially data focusing on valgus knees, are scarce [19].

**Figure 1:**
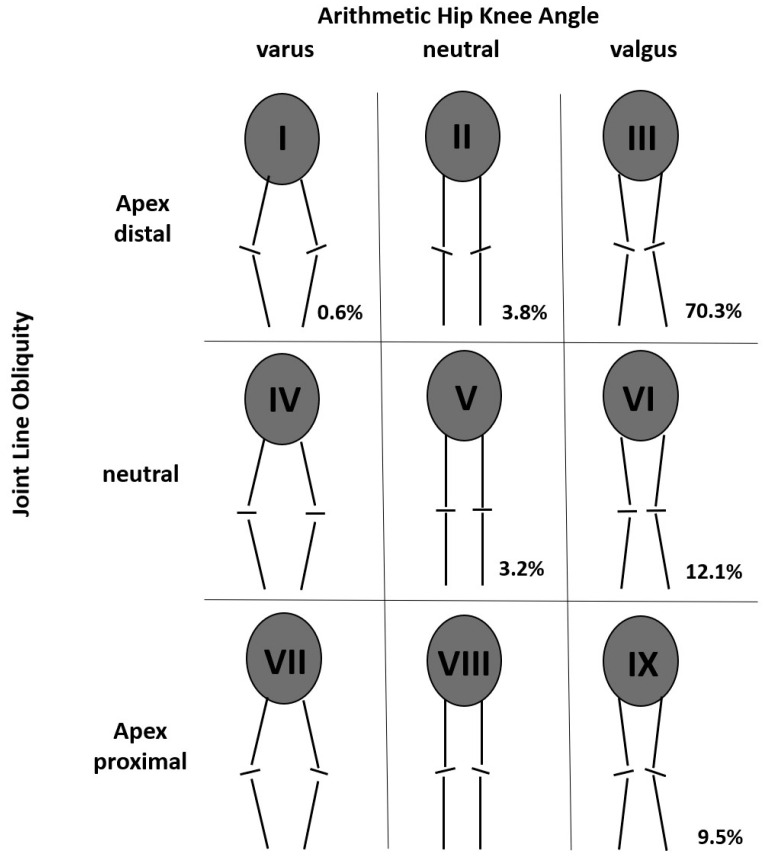
Coronal Alignment of the Knee (CPAK) classification according to MacDessi et al. [21]. Knees are grouped according to arithmetic hip knee angle (varus, neutral, or valgus) and according to the apex of the joint line obliquity (distal, neutral, and proximal). Roman numbers (I–IX) represent group numbers; Arabic numbers display the percentage of the respective group among the study cohort.

A variety of different alignment classification systems and accompanying knee phenotypes have been described over the past years [20,21,22]. The Coronal Plane Alignment of the Knee (CPAK) classification by MacDessi et al. [21] groups knees by both the arithmetic hip knee angle (aHKA) and the joint line obliquity (JLO), resulting in nine possible phenotypes [21]. A graphical representation of the classification can be found in Figure 1.

The current study aims to answer the following research questions: (1) What is the distribution of native knee phenotypes according to CPAK classification in a cohort of knees with valgus osteoarthritis? (2) Does the outcome following MA TKA with standardized soft-tissue release differ depending on the native knee phenotype?

## 2. Materials and Methods

This is a retrospective study of 222 patients with valgus osteoarthritis who underwent primary TKA with a mechanical alignment target and standardized soft-tissue release [14] by the senior author between 2008 and 2013. In total, 64 patients had to be excluded for the following reasons: 39 patients had no minimum 2-year follow up, 2 patients had posttraumatic osteoarthritis, 3 patients had rheumatoid arthritis, 6 patients had incomplete pre-/postoperative hip to ankle X-rays, 4 patients had prior surgeries affecting limb alignment, and 10 patients had prior ipsilateral total hip arthroplasties. Thus, 158 TKA procedures in 135 patients were included for evaluation.

The study comprised 79.7% females and 20.3% males. The right knee was affected in 54.4% and the left knee was affected in 45.6%. The average BMI was 29.6 kg/m^2^ (range 19.7–58.9, SD 7.1 kg/m^2^). The average age at the time of TKA implantation was 67 years (range 40–89, SD 11 years). The mean follow up was 41 months (range 24–94 months, SD 15 months).

### 2.1. Surgical Technique

The distal femoral cut was regularly performed in 5° valgus. Using an extramedullary jig, the tibial cut was carried out at 90° to the axis of the tibia. The posterior slope was set according to the implant manufacturer’s recommendations. The rotation of the femoral component was determined with reference to Whiteside’s line and the transepicondylar axis. A spacer block with alignment rods was used to confirm the cuts. The soft-tissue release included the following steps: (1) horizontal release of the iliotibial band (ITB) at the level of the joint line, (2) posterolateral corner release beginning at the posterior boarder of the ITB to the lateral boarder of the popliteus tendon (which itself is preserved). The release is continued until a “pop” is felt, confirming the completion of the posterolateral corner release. A more detailed description of this surgical technique was published by Boettner et al. in 2016 [14].

In 98.7%, patella resurfacing was performed; all components were implanted using cemented fixation. Cruciate-retaining inserts were implanted in 0.6% of the cases, posterior-stabilized inserts in 81-0% of the cases, and posterior-stabilized plus inserts in 18.4% of the cases.

### 2.2. Clinical Evaluation

Data collection included preoperative and postoperative range of motion (ROM), Western Ontario and McMaster University Osteoarthritis score (WOMAC, on a Likert scale from 0–96 with 0 being best function), University of California Los Angeles activity score (UCLA), short form-12 (SF-12) score with the components “mental component summary” and “physical component summary”, and pain on a visual analogue scale (VAS) from 0 (no pain) to 10 (worst imaginable pain).

### 2.3. Radiographic Evaluation

Measurements were performed on weight-bearing anterior–posterior hip-to-ankle radiographs. Radiographs were taken before and after surgery.

Hip-to-knee angle (HKA), lateral distal femoral angle (LDFA), and medial proximal tibial angle (prior to TKA)/medial tibial plate angle (after TKA, MPTA) were measured. The arithmetic hip knee angle (aHKA) was calculated, and knees were classified according to the Coronal Plane Alignment of the Knee (CPAK) groups as described by MacDessi et al. [21]. A neutral aHKA was defined as 0° ± 1.5°. Figure 1 displays a graphical representation of the classification.

One investigator repeated measurements of all angles on 30 preoperative and postoperative radiographs to detect intra-rater reliability. Another independent observer measured 30 preoperative and postoperative radiographs to detect inter-rater reliability.

All measurements were obtained in SECTRA PACS software package IDS7 (Sectra AB, Linkoeping, Sweden). The study was approved by the institutional review board at the authors’ institution (IRB number 2020-2282).

### 2.4. Statistical Analysis

Descriptive statistics were performed to describe the means, range, and standard deviation for all variables. The Kolmogorov–Smirnov test was performed to see whether the variables were normally distributed. Levene’s test was used to test for the homogeneity of variables. For metric, normally distributed values, a *t*-test or one-way ANOVA was performed (preoperative WOMAC and SF-12 mental component; changes in WOMAC and SF-12 physical component). For independent, non-parametric variables, a Wilcoxon rank-sum test or the Kruskal–Wallis test was performed (preoperative pain, SF-12 physical and total, UCLA, ROM, changes in SF-12 mental and total, UCLA, ROM, all postoperative PROMs). A binominal test was performed to test the gender distribution in the CPAK groups. Linear regression analysis was performed to test for correlations between metric parameters (changes in alignment angles and PROMs, ROM).

Intraclass correlation coefficient 3.1 (ICC) was used to measure intra- and interrater reliability. Intra-rater reliability was very good for all measurements (ICC range: 0.970–0.998) and inter-rater reliability was good to very good (ICC range: 0.887–0.986).

The statistical analysis was performed for a 95% confidence interval. The significance level was set at α = 0.05.

A standard deviation of 17.6 was previously calculated for WOMAC [14]. A power calculation for an aimed power of 80% with an alpha failure of 0.05 and an effect-size of 0.57 to detect a difference in 10 points showed a required sample size of 112 knees.

The statistical Analysis was performed with SPSS Statistics 22 (IBM, Armonk, New York, NY, USA). The power calculation was performed using G*Power Version 3.1.9.7 (University of Duesseldorf, Duesseldorf, Germany) [23,24].

## 3. Results

### 3.1. Preoperative Alignment

**Figure 2:**
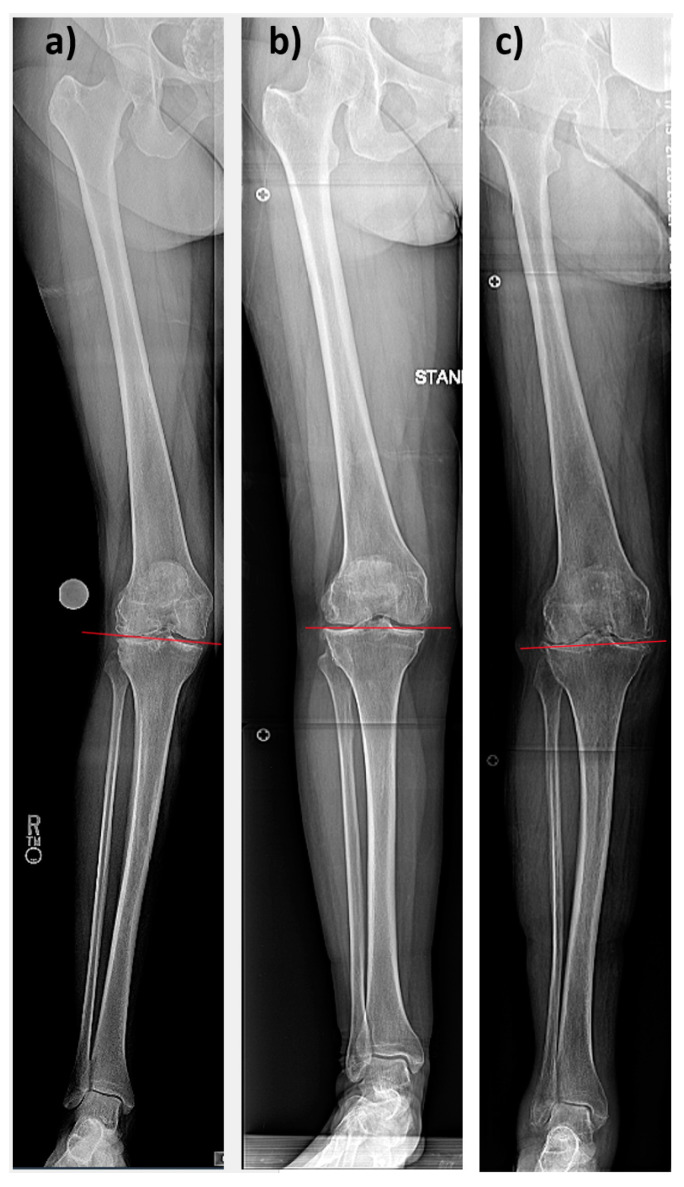
Hip-to-ankle standing radiographs of right knees. All knees are valgus aligned. Red lines schematically show joint line obliquities (JLO) for (**a**) CPAK group III with a distal apex, (**b**) CPAK group VI with a neutral apex, and (**c**) CPAK group IX with a proximal apex.

Table 1 displays the preoperative HKA, LDFA and MPTA. The mean arithmetic HKA (aHKA = MPTA-LDFA) was 5.5° (range −2.5–14.3°, SD 3.1°). This was 3.0° less valgus compared to the actual HKA (range 7–14.3, SD 3.4°). In total, six different CPAK groups were detected. Overall, 70.3% of patients were in group III (valgus aHKA, distal apex of JLO), followed by 12.1% in group VI (valgus aHKA, neutral JLO), 9.5% in group IX (valgus aHKA, proximal apex of JLO), 3.8% in group II (neutral aHKA, distal apex of JLO), 3.2% in group V (neutral aHKA, neutral JLO), and 0.6% in group I (varus aHKA, distal apex of JLO, Figure 1). X-rays exemplifying JLO in the most common groups are presented in Figure 2. The gender distribution differences were significant in group VI (100% females, *p* = 0.007). The other groups did not differ significantly from the overall distribution.

There were no age differences between the groups (*p* = 0.644). The BMI values were higher in group II compared to VI (*p* = 0.033); the other groups showed no differences in BMI. There were no differences in preoperative PROMs and ROM.

### 3.2. Postoperative Alignment and Alignment Chances

**Figure 3:**
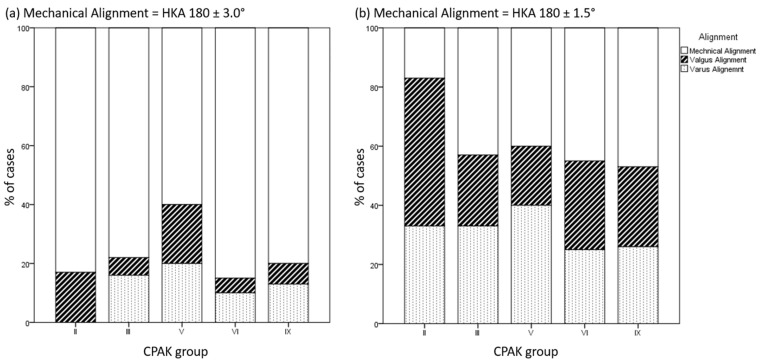
Distribution of postoperative alignment (mechanical alignment: white; valgus alignment: striped; or varus alignment: dotted) in % separately for each CPAK group (*x*-axis): (**a**) if the boundaries for mechanical alignment are set at 180 ± 3° and (**b**) if the boundaries for mechanical alignment are set at 180 ± 1.5° degree.

Table 1 displays the postoperative HKA, LDFA, and MPTA and changes from pre- to postoperative alignment. Overall, 78.5% (124 knees) had a mechanical alignment of HKA 180 ± 3°, and 7.0% (11 knees) had a valgus alignment. If stricter boundaries of 180 ± 1.5° were set, 46.8% (74 knees) were mechanically aligned and 24.7% (39 knees) had a valgus alignment. The postoperative alignment for each CPAK group is shown in Figure 3.

### 3.3. Functional Outcome

The pre- to postoperative improvements were significant for WOMAC, pain, UCLA, SF-12 total and physical score as well as ROM (*p* < 0.001). There was no change in the SF-12 mental component (*p* = 0.688).

**Figure 4:**
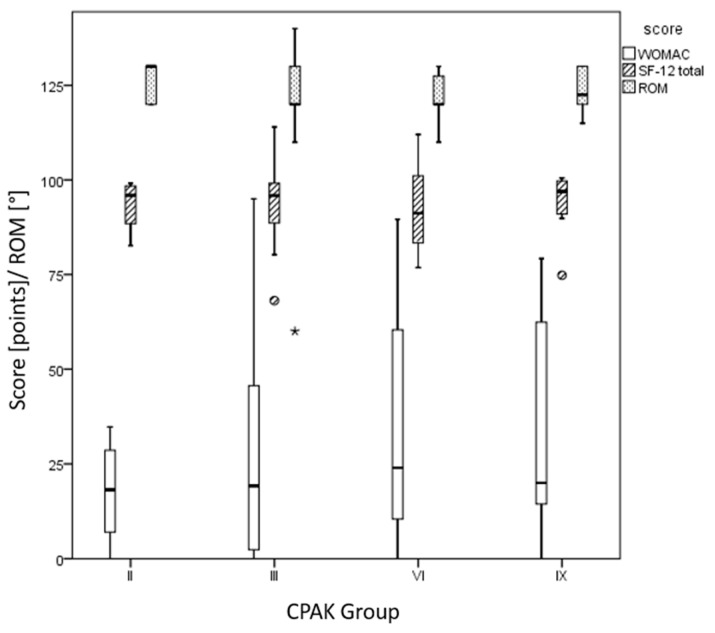
Boxplot diagram showing WOMAC score (in points), SF-12 total score (in points), and range of motion (in degree) of the mechanical aligned knees (*y*-axis) depending on the CPAK group (roman numbers, *x*-axis).

MA aligned TKA (both boundaries ± 3° and ±1.5°) showed no differences in any outcome parameter (postoperative absolute value and improvement compared to the preoperative value for WOMAC, pain, UCLA, all components of SF-12, and ROM) between the CPAK groups. Figure 4 depicts postoperative WOMAC, SF-12, and ROM separately for the CPAK groups.

MA TKA (boundary ± 1.5°) showed better results for postoperative UCLA and improvement in total SF-12 (*p* = 0.011/*p* = 0.028) than TKA with more valgus alignment. Other functional outcomes (pain, the SF-12 mental and physical components, and ROM) showed no differences as displayed in Table 2.

Within CPAK III, MA TKA (boundary ± 1.5°) showed better postoperative UCLA than TKA with valgus alignment (*p* = 0.015). There were no differences for the other parameters or within the other CPAK groups.

Approximately 32.9% (52/158) of the cases had larger valgus deformities with a preoperative HKA > 190°. The deviation between aHKA and postoperative HKA did not correlate with postoperative WOMAC (*p* = 0.835) or ROM (*p* = 0.400).

## 4. Discussion

The current study confirmed a relevant variation in native knee phenotypes even within the subgroup of knees with valgus osteoarthritis. However, the current data suggest that there is no difference in outcome for mechanically aligned TKA between the different phenotypes, implying that mechanical alignment is an appropriate target for the different phenotypes analyzed in the study. In the largest CPAK group, mechanical alignment had more favorable results than leaving the knee in some remaining valgus. If one takes into consideration that mechanical alignment is less likely to result in recurrent knee instability [25], the current study suggests that contrary to findings in varus knees [26,27], phenotype-specific alignment targets might hold fewer benefits for knees with valgus osteoarthritis.

A significant percentage of patients being dissatisfied with their TKA has been reported [28]. Historically, rates have amounted to about 20%; recent studies still report dissatisfaction rates of 10% for all TKAs [29].

Postoperative satisfaction following total joint replacement is multifactorial and includes factors like the patient’s preoperative Kellgren–Lawrence score, socioeconomic status, age, and especially the preoperative psychological stage [29]. Scott et al. found depression, a low preoperative SF-12 mental score, and pain in other joints to be significant risk factors for postoperative dissatisfaction with total knee replacement [30]. A correlation between preoperative anxiety and depression and postoperative dissatisfaction was also reported by Ali et al. in a prospective cohort study [31].

Another factor that has been blamed for pain, instability, and dissatisfaction following TKA is the discrepancy between the native knee alignment and the mechanical alignment targets of TKA [4,9,10,11]. It has been hypothesized that a kinematic aligned knee may feel more “normal” to the patients and make them forget about their replaced joint [28]. It has been further suggested that MA may create gap imbalances that may not be fully correctable with balancing and thus lead to instability [32].

While adequate soft-tissue balancing can be challenging, good results following MA TKA with standardized soft-tissue release have been reported in the literature [14,16,17,33,34].

Ranawat et al. found good results for MA TKA in knees with severe valgus osteoarthritis; none of the 490 knees showed late-onset instability [16]. Boettner et al. found excellent clinical results for MA TKA with standardized soft-tissue release in valgus osteoarthritis with up to 25° valgus deformity of whom only 1% required revision for instability [14]. With a follow-up period up to 11 years, Boyer et al. reported good clinical results and only one revision for septic loosening [33]. Previous studies have further suggested that the degree of preoperative valgus deformity does not impact the outcome of TKA [35].

However, over the past few years, it has been stated that beside the mechanical alignment in the varus and valgus, the joint line obliquity should be taken into account as well [22]. This has led to a variety of classification systems and knee phenotypes that have been published. Mullaji et al. suggested to use nine different groups to describe knees with valgus osteoarthritis [20]. A classification system described by Hirschmann et al. in 2019 is based on ranges of the hip knee angle and the medial tibial and femoral angle; the authors found 43 functional phenotypes in a non-arthritic population and an even wider distribution and higher varus and valgus angles in patients with knee osteoarthritis [22,36]. The CPAK classification as published by MacDessi et al. in 2021 focusses on the native alignment prior to the onset of osteoarthritis. It focuses on the joint line obliquity and uses an arithmetic HKA to estimate the original anatomic phenotype. They described a similar distribution of phenotypes for arthritic and non-arthritic knees [21].

The current study used the CPAK classification to define the different knee phenotypes included in the cohortof knees with valgus osteoarthritis. Even amongst the subgroup of valgus knees, the current data confirmed that there is variation in the native knee phenotypes. The most common phenotype represented by 70% of patients was CPAK group III with a valgus arithmetic hip knee angle and a distal apex of JLO.

However, while there is variance in the alignment phenotypes, one of the crucial questions is whether the existence of these varieties and the general success of a MA TKA are mutually exclusive.

According to the often-criticized difference between the native alignment and the postoperative mechanical axis, one may assume that knees with higher differences may be more likely to have shown worse results. No such correlation was observed in the current study collective. Furthermore, the current data showed significant improvements for all outcome parameters, except the SF-12 mental component, when comparing the pre- to postoperative results.

The second research question aimed to investigate potential differences in outcomes following MA TKA depending on the CPAK type. The current data showed no difference in the outcome for MA TKA with a standardized soft-tissue release between different CPAK groups, thus suggesting that MA TKA is an adequate and successful treatment for knees with valgus osteoarthritis independent of the native alignment type.

It is possible that patients without medial opening, with partially correctable deformities of less than 10 degrees, can be operated on with kinematic alignment. The partial correctability will allow to decrease the overall valgus alignment into a more acceptable range (less than 5 degrees), and since there is no preoperative stretching and weakening of the medial collateral ligament, it is less likely that a knee with mild valgus alignment will become unstable in the future. It has to be taken into consideration that any valgus momentum while loading the knee will increase the risk of medial collateral ligament injury [37].

Patients with larger deformities and considerable medial opening or medial instability, however, are unlikely to benefit from kinematic alignment. By definition, taking the stretched out medial collateral ligament into consideration and trying to avoid soft-tissue release will: (1) require more significant lateral bone resections of the lateral distal femoral condyle and lateral tibia to accommodate the medial opening when balancing the extension gap, (2) will therefore result in more significant valgus alignment, and (3) will continue to stress the already compromised medial collateral ligament which might result in secondary instability. We also want to stress that many patients with more severe valgus deformities of more than five degrees are bothered by the appearance of their legs. “Doctor, will you straighten my leg?” is a very commonly encountered question when treating patients with valgus deformities. This is less requested by patients with mild varus osteoarthritis, suggesting that “knocked knees” are less desirable cosmetically.

Judging the quality of the medial collateral ligament, the correctability of the deformity, and then performing an individualized soft-tissue release is challenging even for an experienced surgeon. Ranawat et al. have therefore, for many years, recommended to use a three-degree distal femur cut to avoid leaving the knee in valgus and reduce the risk of a recurrent instability [16]. While it is tempting to balance the knee through bony resection, an alternative option is to consider standardized soft-tissue release with the option of a posterior stabilized plus (PS+) insert in case a perfect balance cannot be achieved as has been published in the past. Excellent midterm and long-term results have been reported with this standardized approach [14].

The current study has the following limitations: (1) the study design is retrospective and (2) there is no control group who had undergone TKA with a kinematic/anatomic alignment approach. Therefore, the current study cannot prove that patients in certain CPAK groups would not have had an even better outcome with a different alignment target. However, since the largest group did not show a benefit in restoring the anatomic phenotype, the vast majority of valgus knees (>70%) will likely benefit from mechanical alignment. (3) Due to the uneven distribution to the alignment groups, the case numbers were small for some groups; further studies with larger case numbers are required for more detailed and robust comparisons between the groups.

## 5. Conclusions

While there is variance in the native alignment phenotype of knees with valgus osteoarthritis, the individual knee phenotype in patients with valgus osteoarthritis did not influence the outcome after mechanical aligned TKA with standardized soft-tissue release in this study. In addition, the current data suggest that phenotype reconstruction for valgus knees may be less beneficial and that mechanical aligned TKA is an adequate technique to treat knees with valgus osteoarthritis and remains the gold standard independent of the native knee phenotype.

## Figures and Tables

**Table 1:** Pre- and postoperative alignment angles. Mean, range, and standard deviation of pre- and postoperative hip knee angle (HKA), lateral distal femoral angle (LDFA), and medial proximal tibial angle (MPTA) as well as changes (Δ) in these angles.

	Mean [°]	Range [°]	Standard Deviation [°]
Preoperative			
HKA	187.4	181.6–197.7	3.9
LDFA	85.4	81.4–89.9	2.1
MPTA	90.4	84.6–98.0	2.6
Postoperative			
HKA	179.6	172.8–187.3	2.5
LDFA	90.9	84.9–96.1	2.0
MPTA	90.4	86.8–93.1	1.4
Changes pre- to postoperative			
ΔHKA	8.9	−1.8- 31.3	5.9
ΔLDFA	−5.8	−20.7–−0.1	2.9
ΔMPTA	0.2	−7.0–11.4	3.1

**Table 2:** Range of motion (ROM) and patient-reported outcomes following knee arthroplasty. Postoperative WOMAC, SF-12, and UCLA scores, pain (NRS from 1–10), and range of motion as well as changes (Δ) in these outcomes from the pre- to postoperative period.

Outcome	Valgus Alignment	Mechanical Alignment	*p*
WOMAC [points]	30 (0–88), SD 26	28 (0–95), SD 28	0.506
SF-12 physical component [points]	46 (19–63), SD 8	47 (33–59) SD 7	0.380
SF-12 mental component [points]	47 (45–58), SD 6	47 (33–59), SD 6	0.649
SF-12 total [points]	93 (75–112), SD 8	94 (68–114), SD 8	0.422
UCLA [points]	5 (1–10), SD 2	6 (2–10), SD 2	0.011 *
Pain [points]	2 (0–8), SD 2	2 (0–8), SD 2	0.914
ROM [degree]	123 (60–140), SD 9	124 (110–140), SD 6	0.586
ΔWOMAC [points]	−18 (−69–54), SD 29	−25 (−77–47), SD 27	0.106
ΔSF-12 physical component [points]	6 (−10–26), SD 8	9 (−4–23), ‚SD 6	0.463
ΔSF-12 mental component [points]	0 (−26–13), SD 8	0 (−26–18), SD 9	0.849
ΔSF-12 total [points]	6 (−11–29), SD 8	9 (−5–27), SD 7	0.028 *
ΔUCLA [points]	1 (−6–8), SD 2	1 (−2–6), SD 2	0.246
ΔPain [points]	−6 (−10–5), SD 3	−5 (−10–3), SD 2	0.358
ΔROM [degree]	12 (−15–85), SD 16	8 (−30–60), SD 15	0.910

* Significant difference between mechanical and valgus aligned TKA (significance level set at α = 0.050).

## Data Availability

The data presented in this study are available from the corresponding author upon reasonable request. The data are not publicly available due to ethical restrictions.
